# Exome sequencing in unspecific intellectual disability and rare disorders

**DOI:** 10.1186/1755-8166-7-S1-I26

**Published:** 2014-01-21

**Authors:** Anita Rauch

**Affiliations:** 1 University of Zurich, Institute of Medical Genetics, Schlieren-Zurich, Switzerland

## 

Identification of disease causing mutations in genetically heterogeneous conditions such as intellectual disability by Sanger sequencing is time-consuming, costly and often unsuccessful. The advent of NGS techniques is paving the way for novel large scale approaches with an unforeseen diagnostic power. However, the plethora of variants of unknown significance detected by genome-wide approaches requires distinctive strategies to identify actually disease-related mutations. We recently showed that exome sequencing of patient-parent trios in sporadic cases of unspecific severe intellectual disability may unravel disease causing mutation in more than 50% of previously unsolved cases. Thereby it became also evident, that the current descriptions of phenotypes associated with mutations in a certain gene, are heavily biased towards certain recognizable patterns. However, while whole exome sequencing may currently provide theoretically the highest cost-efficient diagnostic power, it may miss mutations due to incomplete coverage of certain genes. Therefore in some phenotypes a “clinical exome” limited to a set of genes with currently known monogenic mutations may also be useful.

